# Adult patent Ductus Arteriosus complicated by endocarditis and hemolytic anemia

**Published:** 2015-06-30

**Authors:** Feridoun Sabzi, Reza Faraji

**Affiliations:** 1 Preventive Cardiovascular Research Centre Kermanshah, Kermanshah University of Medical Sciences, Kermanshah, Iran; 2 Yazd Cardiovascular Research Center, Shahid Sadoughi University of Medical Sciences, Yazd, Iran

**Keywords:** Ductus Arteriosus, endocarditis, hemolytic, anemia, fatigue, dyspnea, leukocytosis

## Abstract

An adult with a large patent ductus arteriosus may present with fatigue, dyspnea or palpitations or in rare presentation with endocarditis. The case illustrated unique role of vegetation of endocarditis in hemolytic anemia in adult with patent ductus arteriosus (PDA). Despite treatment of endocarditis with complete course of appropriate antibiotic therapy and normality of C- reactive protein, erythrocyte sedimentation rate and leukocytosis and wellness of general condition, transthoracic echocardiography revealed large vegetation in PDA lumen, surgical closure of PDA completely relieved hemolysis, and fragmented red cell disappeared from peripheral blood smear. The 3-month follow-up revealed complete occlusion of PDA and abolishment of hemolytic anemia confirmed by clinical and laboratory examination.

## Abstract (es)

Un adulto con un gran ductus arterioso permeable puede presentar fatiga, disnea y palpitaciones y menos frecuentemente presentar endocarditis. El caso muestra el papel de la vegetación de la endocarditis en la anemia hemolítica con el conducto arterioso patente (CAP) en adultos. A pesar del tratamiento de la endocarditis con la terapia antibiótica completa, la normalidad en la proteína C-reactiva, la tasa de sedimentación globular y leucocitaria, y un estado de bienestar general del paciente, la ecocardiografía trans torácica reveló gran vegetación en el lumen de CAP y el cierre completo quirúrgico de PDA, sin hemólisis y la desaparición de glóbulos rojos fragmentados en frotis de sangre periférica. Los 3 meses de seguimiento revelaron oclusión completa de CAP y la desaparición de la anemia hemolítica confirmada por examen clínico y laboratorio.

## Introduction

The clinical spectrum of presentation of a Patent Ductus Arteriosus (PDA) in adult may range from no sign and symptom which is incidentally found on routine physical exam or Transthoracic Echocardiogram (TTE) for other purposes, to patients who present with dyspnea, cardiac failure, pulmonary hypertension, endocarditis, atrial fibrillation, or pneumonia 1. Concomitant clinical presentation of PDA endocarditis with hemolytic anemia due to collision and sheer stress of blood across of PDA that narrowed by vegetation is exceedingly rare phenomenon. It is a clinical consensus of opinion experts that PDA with or without evidence of volume overload should be closed, but less evidence exists concerning the PDA induced hemolytic anemia 2. To the best of our knowledge, this is the first case of hemolytic anemia reported in adult with PDA complicated by endocarditis. We think that endocarditis caused serration of PDA lumen by attached rough vegetation. The pathophysiology is proposed to be the same as that of paravalvar leaks. Hemolytic anemia provoked by turbulent blood flow across the PDA, which its endothelium was damage by endocarditis and vegetation. Intra luminal vegetation with rough surface plays as mechanical barrier and caused turbulent flow and hemolytic anemia 3. Even though, native PDA endocarditis is well known, there are limited data on vegetation-related hemolysis. The prevalence of a hemolysis after PDA endocarditis is unknown. This case, reports the first infective endocarditis on a PDA complicated with hemolysis and highlights the need for proper diagnosis and treatment. Even though hemolysis was eliminated by PDA ligation but further evaluation, and follow-up with TEE revealed no residual shunt or sequel of endocarditis.

## Case description

We report the case of a 28-year-old woman with endocarditis of PDA containing large vegetation that complicated with hemolytic anemia referred from a general hospital to our center. Physical examination showed blood pressure of 110/70 mm Hg, pulse of 92 bpm, temperature of 38.5 °C, and her skin had yellow appearance, the patient noticed 'brown colored urine' and eye exam reveals sclera jaundice. Laboratory data also indicated severe hemolysis and fragmentation of red blood cell (RBC) requiring blood transfusion. Hemoglobin was 9 g/dL, free hemoglobin 60 mg/dL, hematocrit 30%, relative reticulocyte count 9%, haptoglobin 7 mg/dL, total bilirubin 2.9 mg/dL, lactate dehydrogenase (LDH) 1,800 (IU/L). Three blood cultures showed *Streptococcus pneumoniae*.

A bone marrow aspiration was performed to rule out the possibility of an underlying hematological malignancy, and it revealed mildly increased erythropoiesis but no changes indicative of a hematological malignancy were observed. Direct coombs test was positive. *Mycoplasma pneumoniae* IgM antibody was negative. Urinalysis was strongly positive for both hemoglobin and RBC. Further extensive studies excluded immunological and infectious causes of RBC destruction, and cold agglutinin were negative. Thrombophilia tests and G6PD were normal. The patient has not received hepatotoxic drugs or hemolytic drugs.

**Figure 1.**
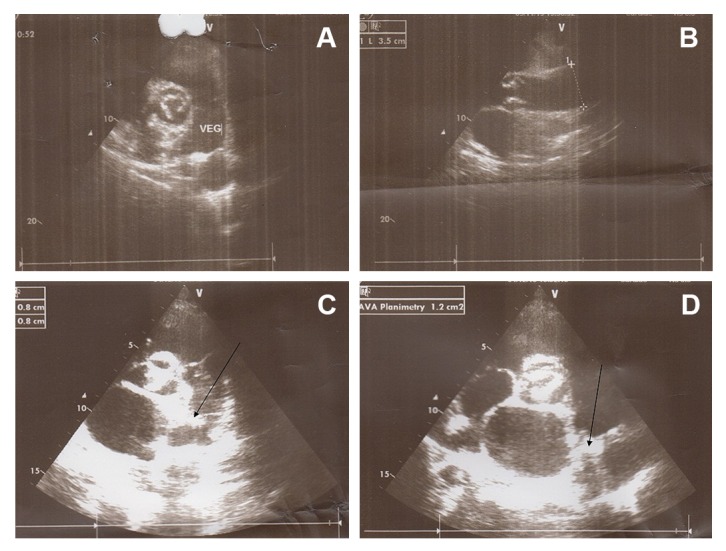
**A. **Vegetation in PDA extended to main pulmonary artery. **B.** Concomitant aortic valve calcification with large aortic diameter due to left to right shunt. **C.** Revealed vegetation in pulmonary side of PDA. **D.** Shows vegetation in pulmonary side of PDA.

In physical examination, the cardiac examination was not able for prominent grade 3/6 systolic murmurs best heard at the left upper sternal border in second inter costal space with radiation into the anterior left chest. Echocardiogram revealed the presence of a PDA in the setting of normal left ventricular function. However, it had a large diameter but a small shunt was not on TTE echocardiography that caused hemolysis. Transthoracic Echocardiogram showed the left ventricle with conserved systolic function, mild aortic insufficiency with calcified valve, and a fixed structure on the wall of the pulmonary artery with erratic movement indicative of vegetation on orifice of PDA (Figs. 1A and B). Transthoracic Echocardiogram suggested a large (10 mm) PDA with left to right shunt. Pulmonary artery mean pressure was 36 mm Hg, consistent with mild pulmonary hypertension (25-35 mm Hg). Moderate left and right atrial enlargement and a mildly enlarged main pulmonary artery were noted.

Before admission, she presented with recurrent lung infection that required antibiotic treatment. Chest radiography showed a prominent main pulmonary artery, aorta and haziness on left upper lung field indicating treated septic emboli. Treatment with appropriate antibiotics was started and continued for four weeks. Fever and patents general condition improved with antibiotics but her jaundice was continued (total bilirubin 3.4 mg/dL). After a complete course of antibiotics therapy, laboratory workup showed C-reactive protein 33 mg/L, leukocytes 12,000/mm^3^, hemoglobin 8.7 g/dL, hematocrit 32%, fragmented red cell on blood smear, negative combs test, which suggested persistent hemolysis despite of recovery from infection. With antibiotic therapy blood cultures became negative for *Streptococcus pneumoniae*. A hypothesis of hemolysis at PDA tract was confirmed by TEE, which showed large intra luminal vegetation on the main pulmonary artery side of PDA (Figs. 1C and D). With continuation of hemolysis and no regression of intra luminal vegetation, surgical ligation of the PDA was undertaken. The thorax was entered through the fourth intercostal space. The pericardium was opened, and the PDA was identified, dissected, and ligated. After surgical closure of PDA, hemolytic anemia resolved completely after PDA closure. Organized non-mobile vegetation reduced risk of emboli during surgical ligation of PDA. Six month follow up revealed disappearance of jaundice and TEE showed complete closure of PDA with no any shunt.

The mechanism of intravascular hemolysis due to PDA endocarditis with its turbulent flow is not described obviously in medical literature. Rapid acceleration, fragmentation and collision of high-velocity blood across of vegetation are associated with a high shear stress that leads to hemolysis. In vegetation-induced hemolysis in PDA, a distinct patterns of turbulent flow that were associated with high shear stress, would thought to disclose the etiology of the hemolysis 4,5. Hemolysis with PDA endocarditis is associated with presence of turbulent ductal flow 6. The likely mechanism of hemolysis is high-velocity turbulent blood flow past the ductal vegetation; leading to mechanical fragmentation of erythrocytes 7. There is no case report of an adult patient with a PDA who developed severe intravascular hemolysis after endocarditis. This hemolysis related to shunting and collision of red blood cell across the vegetation. The case illustrated unique role of vegetation like of Amplatzer device in hemolytic anemia after PDA closure. Device closure of any septal defects may result in hemolysis. Godart reported that hemolysis has been documented after Amplatzer device closure of PDA 8,9. Lambert found that device closure of ASD would be associated with hemolysis 10. Spence also reported this complication after device cloture of VSD 11. In Sivakumar study hemolysis has also reported with the use of devices to close paravalvar mitral valve leaks after mitral valve replacement 12. This report represents the rare case of hemolytic anemia requiring surgical closure of PDA. High turbulent shear stresses are present at locations with high-velocity gradients and at locations immediately distal to vegetation. The flow becomes more disturbed as it travels further across the PDA 13.

## Conclusion

Complications of PDA endocarditis with hemolysis are exceedingly rare. We describe refractory intravascular hemolysis due to a turbulent effect of intra PDA vegetation, a rare complication of PDA endocarditis.
